# Correlative Light-Electron Microscopy detects lipopolysaccharide and its association with fibrin fibres in Parkinson’s Disease, Alzheimer’s Disease and Type 2 Diabetes Mellitus

**DOI:** 10.1038/s41598-018-35009-y

**Published:** 2018-11-14

**Authors:** Greta M. de Waal, Lize Engelbrecht, Tanja Davis, Willem J. S. de Villiers, Douglas B. Kell, Etheresia Pretorius

**Affiliations:** 1 Department of Physiological Sciences, Stellenbosch University, Stellenbosch, Private Bag X1 Matieland, 7602 South Africa; 2 Central Analytical Facilities, Fluorescence Microscopy Unit, Stellenbosch University, Stellenbosch, Private Bag X1 Matieland, 7602 South Africa; 3 Department of Internal Medicine, Stellenbosch University, Stellenbosch, Private Bag X1 Matieland, 7602 South Africa; 4 School of Chemistry, The University of Manchester, 131 Princess St, Manchester, Lancs M1 7DN UK; 5 Manchester Institute of Biotechnology, The University of Manchester, 131 Princess St, Manchester, Lancs M1 7DN UK

## Abstract

Many chronic diseases, including those classified as cardiovascular, neurodegenerative, or autoimmune, are characterized by persistent inflammation. The origin of this inflammation is mostly unclear, but it is typically mediated by inflammatory biomarkers, such as cytokines, and affected by both environmental and genetic factors. Recently circulating bacterial inflammagens such as lipopolysaccharide (LPS) have been implicated. We used a highly selective mouse monoclonal antibody to detect bacterial LPS in whole blood and/or platelet poor plasma of individuals with Parkinson’s Disease, Alzheimer’s type dementia, or Type 2 Diabetes Mellitus. Our results showed that staining is significantly enhanced (P < 0.0001) compared to healthy controls. Aberrant blood clots in these patient groups are characterized by amyloid formation as shown by the amyloid-selective stains thioflavin T and Amytracker™ 480 or 680. Correlative Light-Electron Microscopy (CLEM) illustrated that the LPS antibody staining is located in the same places as where amyloid fibrils may be observed. These data are consistent with the Iron Dysregulation and Dormant Microbes (IDDM) hypothesis in which bacterial inflammagens such as LPS are responsible for anomalous blood clotting as part of the aetiology of these chronic inflammatory diseases.

## Introduction

Many chronic diseases, including those classified as autoimmune, cardiovascular, or neurodegenerative, are associated with persistent inflammation. Although typically mediated by ‘inflammatory’ cytokines and affected by both environmental and genetic factors, the origin of this inflammation is mostly unclear. Since the recognition that most peptic ulcers, other than those caused by non-steroidal anti-inflammatory drugs (NSAIDs), have a microbial basis^1,2^, there is a significant body of literature that suggests many other supposedly non-communicable diseases might actually have a bacterial and/or viral origin. For instance, *Herpes simplex* virus type 1 (HSV1), *Chlamydia pneumoniae*, and several types of spirochaetes are specific microbes that have been implicated in the aetiology of Alzheimer’s Disease (AD)^3–14^. A bacterial link has also been suggested for Parkinson’s Disease (PD)^3,15–21^, and microbes have been associated with ageing in general^22^. The presence of an aberrant blood microbiome, as assessed by sequencing, has also been implicated in Type 2 Diabetes (T2D) and cardiovascular events^23–25^.

These microbes are not detected by standard microbiological tests involving replicative culture because they are dormant (e.g.^26–30^). The exit from dormancy has been linked to dysregulation of iron metabolism, and/or stress hormones^31–33^, in part because free iron is required for the reactivation and/or growth of the microbes in question. This reactivation of low levels of bacteria can then release highly potent inflammagens such as lipopolysaccharide (LPS) from Gram-negative organisms and lipoteichoic acids (LTA) from Gram-positive organisms. Therefore, in a series of papers, as summarised in a review^30^, we refer to these dormancy/iron/inflammation events as the Iron Dysregulation and Dormant Microbes hypothesis (IDDM). In this hypothesis we argue that microorganisms and their circulating products represent an important external stimulus in inflammatory conditions such as PD, AD, T2D as well as in other diseases such as pre-eclampsia^34,35^, overlaid on any genetic disease predisposition and exposure to environmental stressors.

LPS and LTA have recently been shown to be capable of triggering hypercoagulation or aberrant blood clotting into an amyloid form^36–39^. Hypercoagulation is a well-known hallmark of inflammation^40^ and is caused by pathological levels of circulating inflammatory molecules, including pro-inflammatory cytokines^36^. Importantly, during inflammation, erythrocytes (RBCs) and platelets are also involved in the pathological clotting process, where circulating mediators of inflammation cause the membranes of these cells to become altered into procoagulant surfaces^41–46^. Also, it was recently reported that von Willebrand factor plays a role in erythrocyte endothelial adhesion, where eryptotic erythrocytes may interact with von Willebrand factor fibres^47^. In inflammatory conditions, including PD, AD and T2D, a changed erythrocyte and platelet structure, together with close interactions with pathological fibrin (atypical fibrin fibre formation), were previously reported^48–51^. Such a hypercoagulable state and anomalous blood clotting go hand in hand^37,52,53^, resulting in pathological clotting, and is one of the main causes contributing to myocardial infarction and thrombo-embolic strokes^54,55^. Therefore these bacterial inflammagens (LPS and LTA) may have a much larger role in inflammatory conditions, such as PD, AD, T2D and others, than is presently conceived.

Specifically, we have shown that the addition of minute concentrations (highly substoichiometric amounts (10^−8^ molar ratio)) of LPS and LTA to healthy blood plasma, together with thrombin to create a clot, can result in fibrin(ogen) plasma proteins adopting an amyloid form^37–39^. We also described the reversal of this aberrant clotting by the addition of LPS-binding protein to healthy blood. Similarly, we have shown blood clots with an amyloid form in PD, AD and T2D^37,39,49,56–59^. We could also reverse this amyloid state, by adding LPS-binding protein to plasma of these patients^37,39,49,56–59^. This is consistent with the view that LPS and/or LTA have a causal role in these diseases.

**Figure 1:**
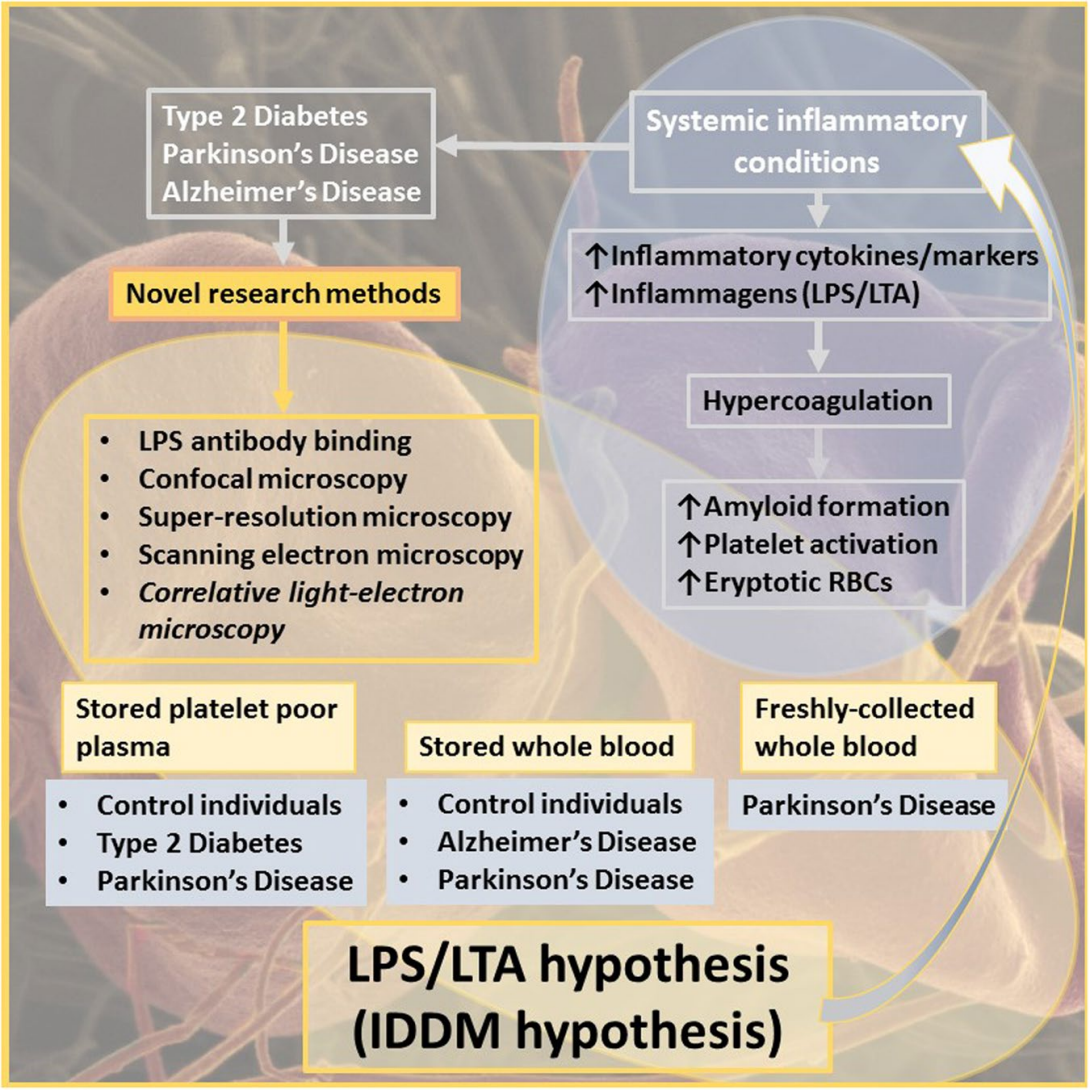
Overview of this paper, focusing on systemic inflammation in various inflammatory conditions, the presence of inflammagens such as LPS, and its contribution to hypercoagulation and amyloid formation, along with a list of novel research methods employed.

The current ELISA methods to determine LPS concentration, based on a *Limulus* amoebocyte lysate or its recombinant factor C^60^, have limitations, as the results can be variable, not least since LPS in plasma is bound to proteins such as apoE^61^. We therefore investigated a novel fluorescence antibody-based technique to detect and measure the levels or amount of LPS in blood. The current paper focuses on developing this method, by first adding various concentrations of LPS to healthy platelet poor plasma (PPP), enriched in fibrinogen, followed by fluorescence antibody detection. After we optimized this method on healthy PPP with added LPS, we used our technique to detect LPS in PPP and/or whole blood (WB) samples of PD, AD and T2D individuals. We used confocal microscopy and super-resolution structured illumination microscopy (SR-SIM) to visualize antibody binding. Furthermore, we used a novel technique, referred to as correlative light-electron microscopy (CLEM), where samples are imaged separately, first using the fluorescence microscopy modality (confocal or super-resolution), and then using a Shuttle and Find functionality, imaging exactly the same area using a high-resolution scanning electron microscope (SEM)^62–64^. We could thereby detect an increased presence of LPS in the blood of individuals with PD, AD and T2D, compared to that of healthy individuals. Furthermore, in this paper, Amytracker™ and thioflavin T (ThT) were used to confirm amyloid formation in PD. Previously, we showed amyloid formation with these fluorescent stains in T2D (refer to the link included in the paper to access raw data: https://1drv.ms/f/s!AgoCOmY3bkKHvEigbzhPJ-gPv1Vr)^58^. We conclude here that this amyloid formation in fibrin(ogen) is, at least in part, a major consequence of the presence of circulating LPS. The corollary of this is that if one could remove or decrease the levels of the circulating LPS, the attendant coagulopathies and hence severity of these diseases might be ameliorated. Figure 1 gives an overview of this paper.

## Materials and Methods

### Ethical statement

Ethical clearance for the collection of blood from all individuals was obtained from the Health Sciences Ethical Committee of the University of Pretoria, as well as from the Health Research Ethics Committee (HREC) of Stellenbosch University (ethical numbers: 80/2013 and reapproved 2015; 81/2013 and reapproved 2015; 298/2016; 1952 and 6592). Written informed consent was obtained from all individuals (available on request). The methods were carried out in accordance with the approved guidelines. Blood was collected and methods were carried out in accordance with the relevant guidelines of the ethics committee. We adhered strictly to the Declaration of Helsinki.

### Sample population

Healthy individuals without known inflammatory conditions and individuals diagnosed with Parkinson’s Disease (PD), Alzheimer’s Disease (AD) and Type 2 Diabetes (T2D) were included in this study. The exclusion criteria for the healthy population included inflammatory conditions such as asthma, smoking, and (if female) being on contraceptive or hormone replacement treatment. These individuals did not use chronic medication nor take any anti-inflammatory medication. The PD individuals were diagnosed by a neurologist with the use of the Unified Parkinson’s Disease Rating Scale (UPDRS)^65,66^. The AD patients were also diagnosed by a neurologist and individuals with vascular dementia were excluded. Exclusion criteria for the individuals with the various inflammatory conditions were smoking and the use of contraceptives or hormone replacement treatment (if female). Conditions including asthma, human immunodeficiency virus (HIV) and tuberculosis also formed part of the exclusion criteria.

### Sample preparation

Whole blood (WB) from ten healthy individuals, eleven individuals diagnosed with PD and ten individuals diagnosed with AD were included in this study. WB was either frozen at −80 °C or used at room temperature, directly after collection. In addition, platelet poor plasma (PPP) was prepared from eleven healthy individuals, eleven individuals diagnosed with PD and ten individuals diagnosed with T2D. WB of all the participants was collected in citrate tubes. PPP was prepared by centrifuging the citrated blood samples at 3000 *g* for 15 minutes at room temperature (±21 °C), followed by storage at −80 °C.

### Confocal microscopy of clots prepared from stored healthy and Parkinson’s Disease (PD) platelet poor plasma (PPP)

Previously, we showed amyloid formation in T2D using the fluorescent stains, thioflavin T (ThT) and Amytracker™ 480 and 680. We also showed amyloid formation in PD, but we only used ThT to show this^58,59^. In this paper, to confirm the presence of amyloid formation in PD PPP, we used ThT as well as Amytracker™ 480 and 680. On the day of analysis, the −80 °C-stored PPPs were brought to room temperature. ThT (final concentration of 5 µM) and Amytracker™ 480 and 680 (final exposure concentration: 0.1 μL stock solution into 100 μL PPP) were added to the healthy and PD PPP samples, followed by incubation for 30 minutes (protected from light) at room temperature. A small volume (10 µl) of the stained PPP sample was transferred to a microscope slide, after which thrombin (provided by the South African National Blood Service) was added in the ratio 1:2 (5 µL thrombin: 10 µL PPP) and slightly mixed to form a clot and to create extensive fibrin networks. After 30 seconds a coverslip was placed over the prepared clot, and samples were viewed immediately using a Zeiss LSM 780 with ELYRA PS1 confocal microscope with a Plan-Apochromat 63×/1.4 Oil DIC objective. The following settings were used:

- For ThT: 488 nm excitation laser, and emission measured at 508–570 nm.
- For Amytracker™ 480: 405 nm excitation laser, and emission measured at 478–539 nm.
- For Amytracker™ 680: 561 nm excitation laser, with emission measured at 597–695 nm.

### Optimisation of LPS antibody binding by using platelet poor plasma (PPP) from healthy individuals with added LPS

Optimal concentrations of the primary antibody, Anti-*E*.*coli* LPS antibody [2D7/1] (mouse monoclonal IgG, ab35654, Abcam), and secondary antibody, Goat Anti-Mouse IgG H&L (Alexa Fluor® 488) (ab150113, Abcam), were determined, using PPP smears from a healthy individual exposed to 5 mg.L^−1^ LPS (from *E*. *coli* O111:B4 (Sigma, L2630)). A primary antibody concentration of 1:200 and secondary antibody concentration of 1:200 gave the optimal fluorescence signal. Secondary antibody and non-stained controls were also included.

**Figure 2:**
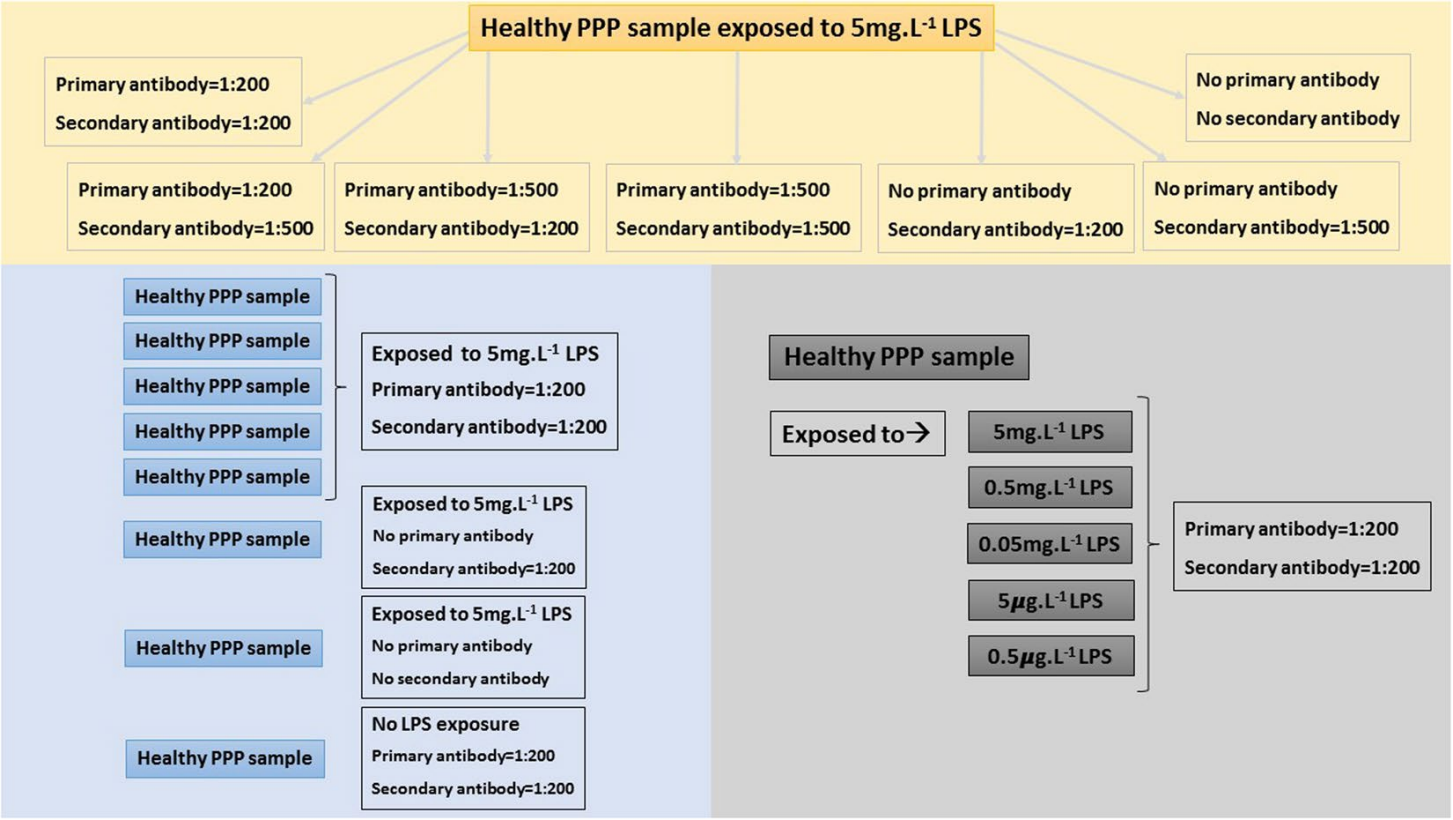
Overview of optimization of LPS antibody binding, using healthy platelet poor plasma (PPP) samples exposed to LPS.

We also prepared PPP smears of five more healthy individuals, all exposed to 5 mg.L^−1^ LPS. Secondary antibody and non-stained controls were also included, as well as a healthy PPP smear with no LPS exposure (negative control). Ultimately, healthy PPP was exposed to decreasing concentrations of LPS, to the point of extinction of any signal. PPP smears from the same healthy individual were exposed to the following LPS concentrations: 5 mg.L^−1^, 0.5 mg.L^−1^, 0.05 mg.L^−1^, 5 ***μ***g.L^−1^ and 0.5 ***μ***g.L^−1^ respectively. See Fig. 2 for an overview of the optimisation of LPS antibody binding.

### Fluorescent detection of LPS in platelet poor plasma (PPP) and whole blood (WB) (freshly collected and/or stored) from healthy individuals and individuals diagnosed with Parkinson’s Disease (PD), Alzheimer’s Disease (AD) and Type 2 Diabetes (T2D)

A smear of either PPP or WB was prepared on a microscope slide, using 5 ***μ***L of PPP or WB. The slide was then air dried for 45 minutes. The sample was fixed with 10% neutral buffered formalin (NBF) for 3–5 minutes, followed by three Gibco^TM^ phosphate-buffered saline (PBS) (pH = 7.4) washes. Next, the sample was blocked with 5% goat serum (prepared in PBS), after which it was stained with primary antibody (1:200 prepared in blocking buffer), Anti-*E*.*coli* LPS antibody [2D7/1] (mouse monoclonal IgG, ab35654, Abcam), for one hour at room temperature. After the sample was again washed three times with PBS, the sample was stained with secondary antibody (1:200 prepared in PBS), Goat Anti-Mouse IgG H&L (Alexa Fluor® 488) (ab150113, Abcam), for one hour at room temperature in the dark. Ultimately, following the last wash step, a coverslip was mounted with a drop of Dako fluorescence mounting medium.

The prepared smears were stored at −20 °C (protected from light), and viewed using a Zeiss LSM 780 with ELYRA PS1 confocal microscope with a Plan-Apochromat 63x/1.4 Oil DIC objective. The 488 nm Argon excitation laser was used, with emission measured with a GaAsP detector at 493–630 nm.

### Correlative light-electron microscopy (CLEM)

CLEM is a novel, albeit expensive and tedious procedure, where a confocal micrograph is correlated on a scanning electron microscope (SEM) micrograph, via a shuttle-and-find system^62–64^. Therefore, we selected stored healthy WB, stored PPP from T2D and PD, as well as freshly-collected PD WB. For CLEM preparation, the same procedure was followed as described above, but a smear of 3 ***μ***L PPP or WB was made on a cover slip instead of a microscope slide. The prepared smear was stored at 4 °C overnight, immersed in double distilled H_2_0.

The coverslip was mounted in the Shuttle and Find Coverslip holder (Zeiss, Germany), which is equipped with a marked coordinate system. Prior to imaging the sample, the microscope is calibrated according to these coordinates, using the Shuttle and Find modality of the ZEN 2012 software (Zeiss, Germany).

For confocal imaging, the microscope setup which was used for quantification remained the same for CLEM. However, for improved resolution and subsequent correlation, the PD WB (fresh) sample was imaged with the super-resolution structured illumination microscopy (SR-SIM) platform. A 488 nm 100 mW laser was for excitation and emission detected with a BP 495–550 filter and captured with an Andor EM-CCD camera iXon DU 885 for SIM. Z-stack micrographs were processed with the ZEN 2012 software, applying an optimised noise filtering algorithm.

Following capturing of the fluorescence micrographs, SEM sample preparation was immediately performed with the cover slip still mounted in the Shuttle and Find Coverslip Holder. Firstly, the sample was fixed with 4% paraformaldehyde in PBS for 30 minutes. The sample was then washed three times (three minutes each) with PBS and incubated with 1% osmium tetroxide in double distilled H_2_0 for 15 minutes. Following fixation, the sample was again washed three times with PBS. The sample was dehydrated using a standard series of ethanol dilutions: 30%, 50%, 70%, 90% and 100% (3x) for three minutes each. The sample was then covered with 99.9% hexamethyldisilazane (HMDS) for 30 minutes to complete sample dehydration. Ultimately, one final drop of HMDS was directly placed onto the sample, after which the sample was left to air dry in a fume hood overnight (±16 hours).

The sample, still mounted in the sample holder, was coated with a thin (~5 nm thick) layer of carbon prior to analysis, using a Quorum Q150T coater by performing carbon rod evaporation. SEM ultrastructural analysis of the selected samples was performed on the Zeiss MERLIN^TM^ field emission scanning electron microscope located in the Central Analytical Facility (CAF) Electron Microbeam Unit, Stellenbosch University. The microscope was first calibrated according to the coordinates of the sample holder after which the areas of interest were located using the Shuttle and Find modality of the ZEN 2012 software. Micrographs were captured with the SmartSEM software (Zeiss, Germany), using high resolution InLens capabilities at 1 kV accelerating voltage of the beam, a working distance of 4.2 mm and a beam current of 86 pA.

After the SEM micrographs were captured, they were overlaid with the fluorescence images with the Shuttle and Find functionality, by identification of three identical points in the SEM and fluorescence micrographs respectively.

### Statistical analysis

Data were analysed and tested for normality using the Shapiro-Wilk normality test. To quantify LPS antibody binding in healthy, PD and T2D PPP, the mean fluorescence intensity of all the images was determined in ImageJ (FIJI)^67^. Analyses were performed using either the Welch’s t test or Mann-Whitney test (depending on the normality of the data), in GraphPad/Prism v7. Statistical significance was accepted at P < 0.05.

### Ethics approval and consent to participate

Ethical clearance was obtained from the Health Sciences Ethical Committee of the University of Pretoria (Ethics References: 80/2013 and reapproved 2015; 81/2013 and reapproved 2015; 298/2016), as well as from the Health Research Ethics Committee (HREC) of Stellenbosch University (Ethics References: 1952 and 6592). A written form of informed consent was obtained from all donors. Blood was collected and methods were carried out in accordance with the relevant guidelines of the ethics committees. We adhered strictly to the Declaration of Helsinki.

## Results

Healthy individuals and individuals diagnosed with PD, AD and T2D were age-correlated and participants included both genders (Table 1). The Hoehn and Yahr scale (used to identify or rate the severity of PD), as well as the HbA1c levels for the individuals diagnosed with T2D are indicated in Table 1.

**Table 1:** Demographics of the individuals included in this study. Data expressed as mean ± SEM.

Sample type	Stored platelet poor plasma		
	Healthy individuals (n = 11)	Type 2 Diabetes individuals (n = 10)	Parkinson’s Disease individuals (n = 11)
Gender	Male (n = 5); Female (n = 6)	Male (n = 5); Female (n = 5)	Male (n = 8); Female (n = 3)
Age (years)	48 ± 3.240 (n = 11)	57.5 [51–67.5]* (n = 10)	71 ± 1.404 (n = 11)
HbA1c (%)		7.4 ± 0.456 (n = 10)	
Hoehn and Yahr scale			2.5 [2.5–3]* (n = 11)
**Sample type**	**Stored whole blood**		
	**Healthy individuals (n = 10)**	**Alzheimer’s Disease individuals (n = 10)**	**Parkinson’s Disease individuals (n = 10)**
Gender	Male (n = 2); Female (n = 8)	Male (n = 7); Female (n = 3)	Male (n = 7); Female (n = 3)
Age (years)	68 ± 5.696 (n = 10)	66 ± 4.740 (n = 10)	71 ± 1.133 (n = 10)
Hoehn and Yahr scale			1.5 (n = 10)
**Sample type**	**Freshly-collected whole blood**		
	**Parkinson’s Disease individual (n = 1)**		
Gender	Female		
Age (years)	69		
Hoehn and Yahr scale	2		

*Data expressed as median and [interquartile ranges].

**Figure 3:**
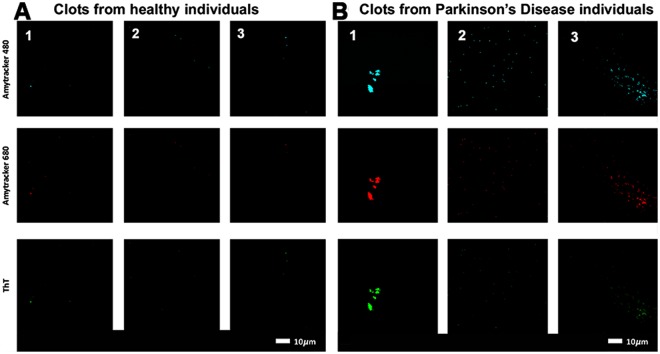
Typical range of confocal micrographs of platelet poor plasma (PPP) with added thrombin, showing the fluorescence amyloid signal of (**A**) healthy individuals and (**B**) Parkinson’s Disease (PD) individuals. Platelet poor plasma (PPP) from each individual was incubated with three specific amyloidogenic fluorescent markers, thioflavin T and Amytracker™ 480 and 680.

The pathologic assembly of amyloidogenic fibrin(ogen) is a direct cause of abnormal clotting or hypercoagulation. We therefore investigated amyloid formation in PPP clots, which reflects pathological clotting. Representative confocal micrographs of (A) clots prepared from PPP of healthy individuals versus (B) clots prepared from PPP of individuals diagnosed with PD are shown in Fig. 3. PPP was incubated with three specific amyloidogenic fluorescent markers, thioflavin T (binding to open hydrophobic areas on fibrin) and Amytracker™ 480 and 680 (staining classical amyloid structures)^68^.

The fluorescence amyloid signal in samples taken from PD individuals, in comparison with those from healthy individuals, was greatly enhanced. This suggests that there is more amyloid when clots are formed with fibrin(ogen) of PD individuals than with fibrin(ogen) of healthy individuals. This was also previously found in fibrin(ogen) of individuals diagnosed with T2D and AD^57–59,69^.

**Figure 4:**
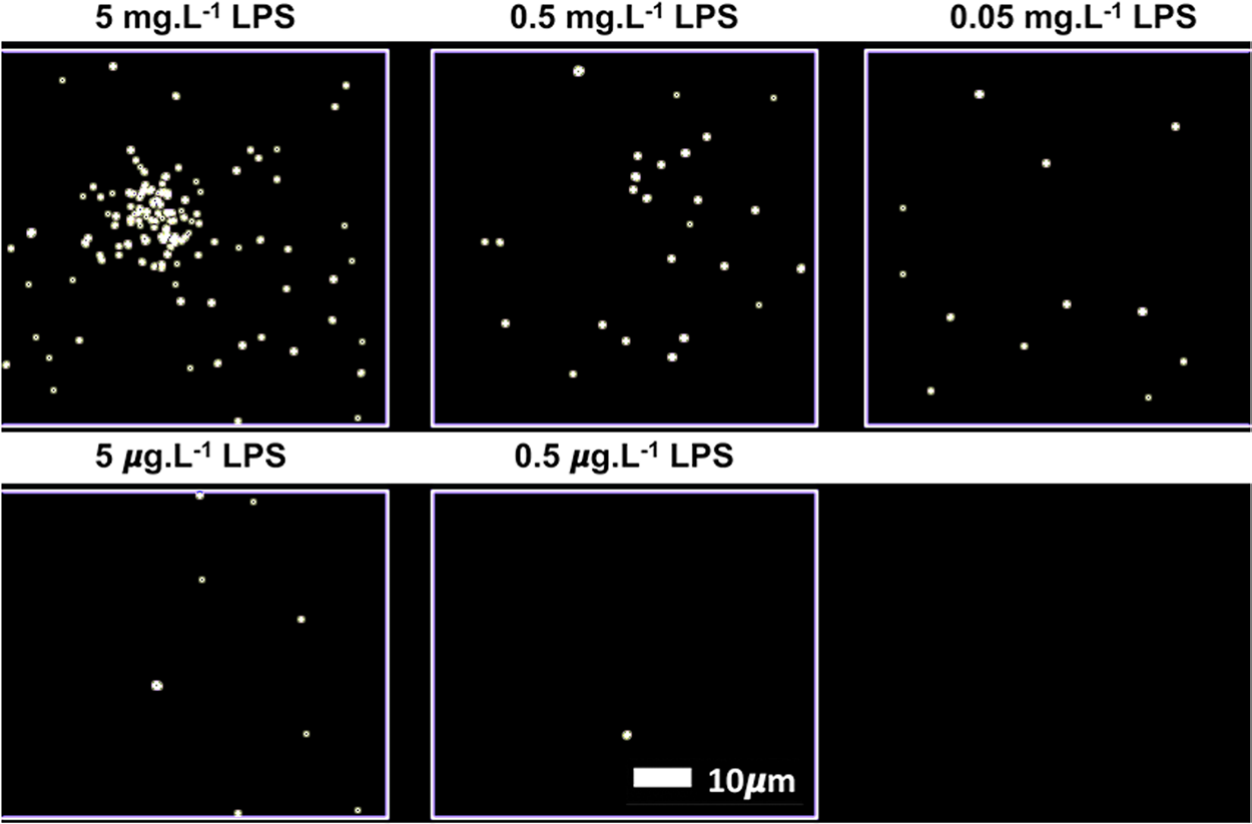
Representative confocal micrographs of healthy platelet poor plasma (PPP) with added LPS to show optimization of detection of LPS, by using anti-*E*.*coli* LPS antibody and a secondary antibody (1:200 dilution for primary and secondary antibodies). We used five different LPS concentrations, and estimated that 0.5 ***μ***gL^−1^ LPS is the lowest detectable concentration. For clarity, we inverted the micrographs, followed by applying the “find edges” function in ImageJ (FIJI), to show the decreasing fluorescence LPS signal with decreasing concentrations added.

**Figure 5:**
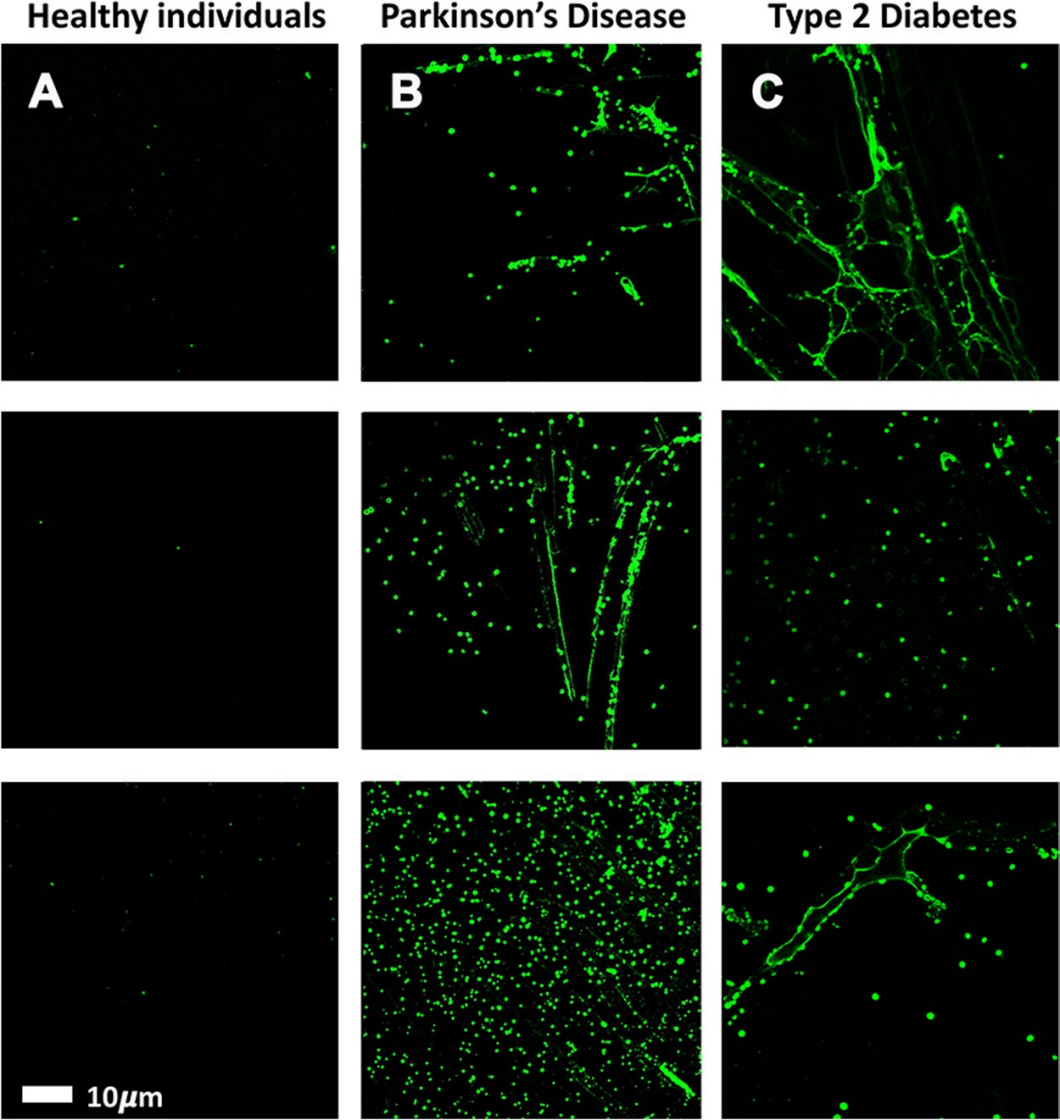
Representative platelet poor plasma (PPP) smears with added anti-*E*.*coli* LPS antibody and secondary antibody, from (**A**) healthy individuals, (**B**) Parkinson’s Disease (PD) individuals and (**C**) Type 2 Diabetes (T2D) individuals (1:200 dilution for primary and secondary antibodies).

**Figure 6:**
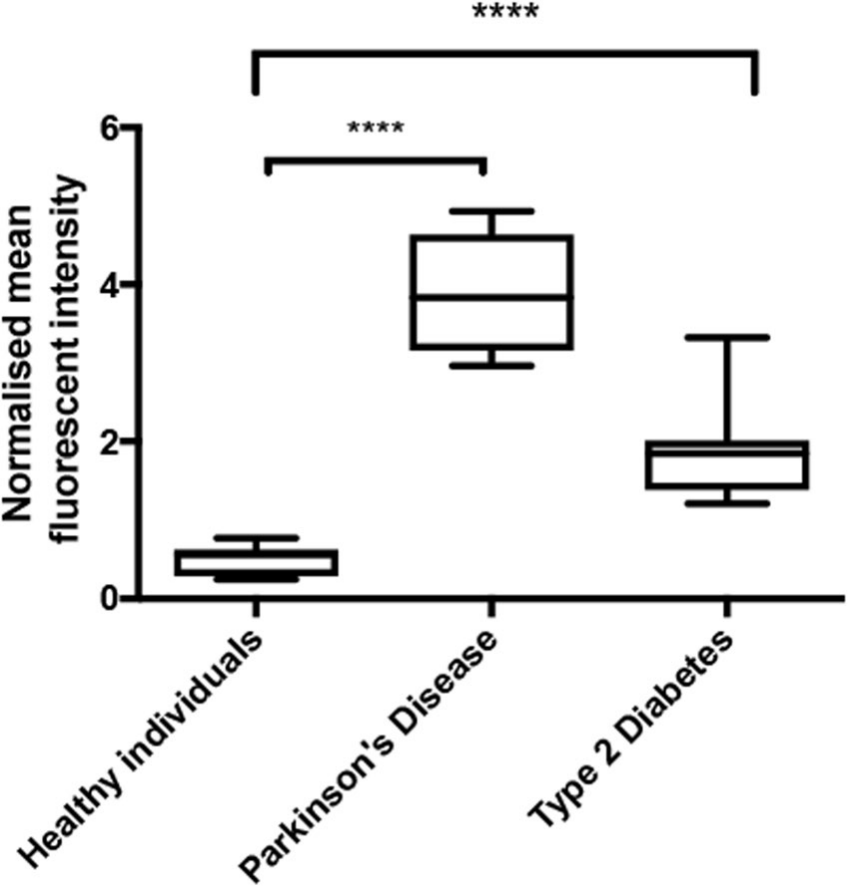
Boxplot of the distribution of the mean fluorescence intensity, normalised by dividing the mean fluorescence intensity values of the healthy individuals, Parkinson’s Disease (PD) individuals and Type 2 Diabetes (T2D) individuals, by the mean fluorescence intensity values of the corresponding secondary antibody control. In this way, we accounted for non-specific secondary antibody binding; ****P < 0.0001.

**Figure 7:**
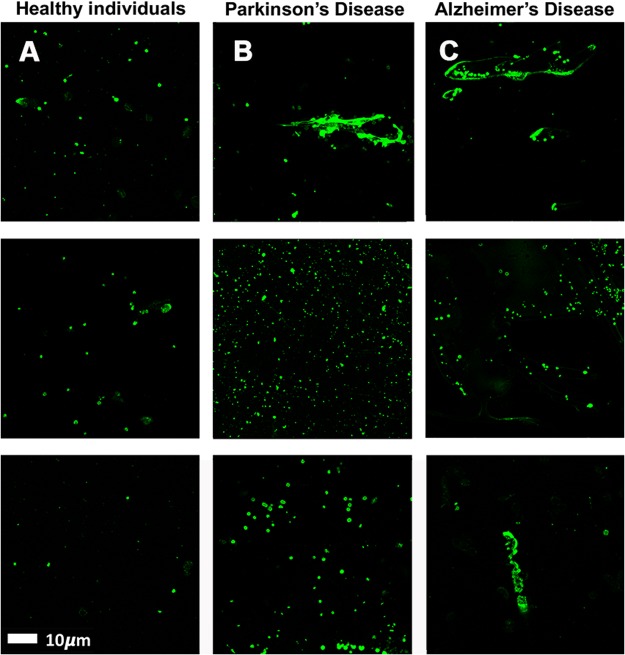
Representative whole blood (WB) smears with added anti-*E*.*coli* LPS antibody and secondary antibody, from (**A**) healthy individuals, (**B**) Parkinson’s Disease (PD) individuals and (**C**) Alzheimer’s Disease (AD) individuals (1:200 dilution for primary and secondary antibodies).

PPP smears from the same healthy individual were exposed to decreasing LPS concentrations. This formed part of optimizing the method for determining the presence of LPS in PPP, via anti-*E*.*coli* LPS antibody and a secondary antibody (see Fig. 4 for examples). So far as we are aware, this has not previously been done in PPP. We studied antibody binding in PPP from healthy individuals, PD individuals and T2D individuals (see Fig. 5 for representative micrographs). Figure 6 illustrates the distribution of the (normalised) mean fluorescence intensity of the confocal micrographs of the healthy, PD and T2D PPP. There is a significant increase in the fluorescence LPS signal in PPP from PD individuals, in comparison with healthy individuals (P value < 0.0001). There is also a significant increase in antibody binding in PPP taken from T2D individuals, in comparison with those of healthy individuals (P value < 0.0001). See Fig. 7 for representative confocal micrographs of antibody binding in WB from healthy individuals, PD individuals and AD individuals. This is a confirmation that LPS can be detected with antibody staining in both PPP and WB samples.

**Figure 8:**
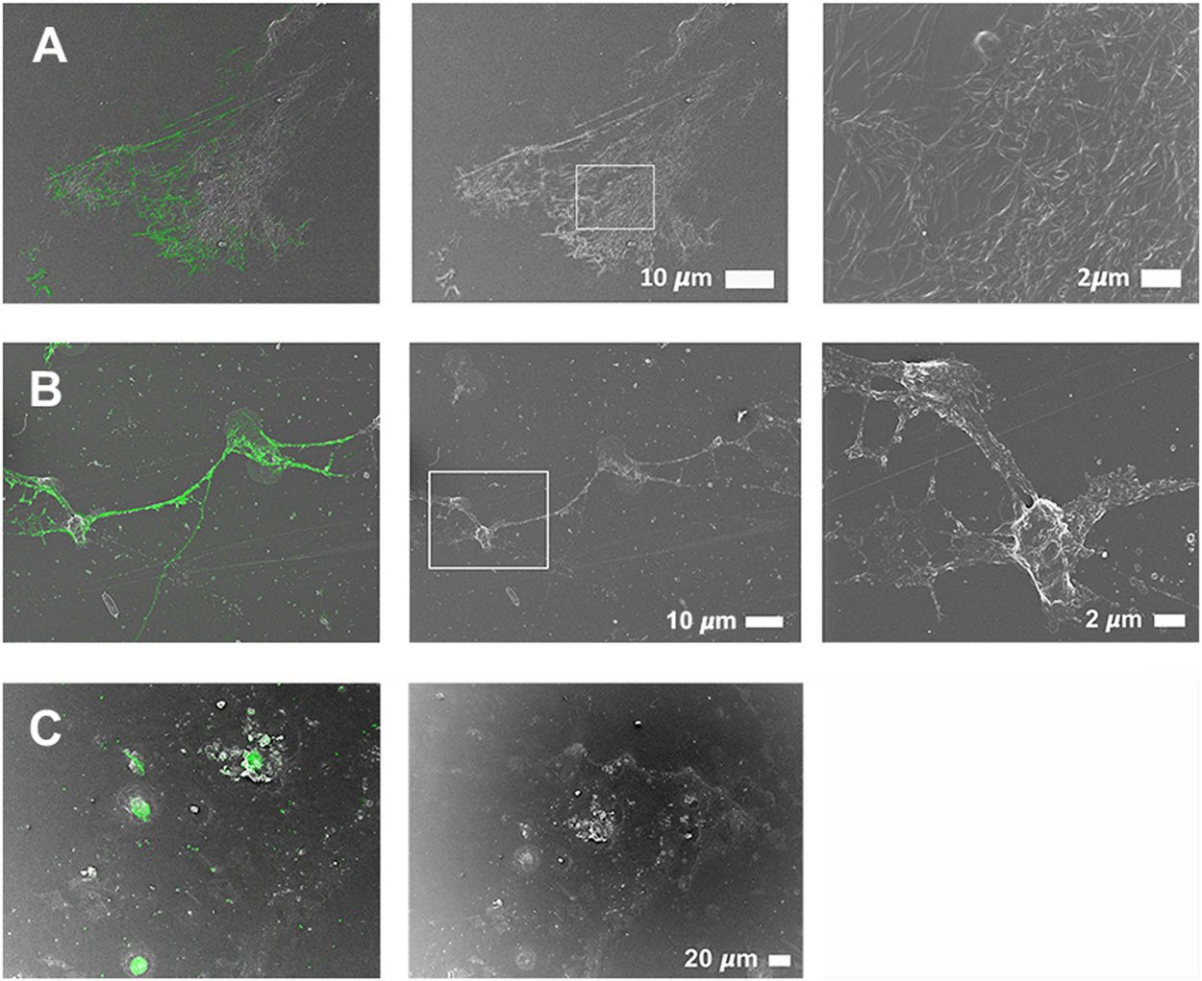
Representative CLEM and SEM micrographs of (**A**) stored platelet poor plasma (PPP) from a Type 2 Diabetes (T2D) individual, (**B**) freshly-collected whole blood (WB) from a Parkinson’s Disease (PD) individual and (**C**) stored whole blood (WB) from a Parkinson’s Disease (PD) individual (1:200 dilution for primary and secondary antibodies). The fluorescence microscopy modalities used were super-resolution (SR-SIM) for (**B**) and confocal for (**A**) and (**C**).

**Figure 9:**
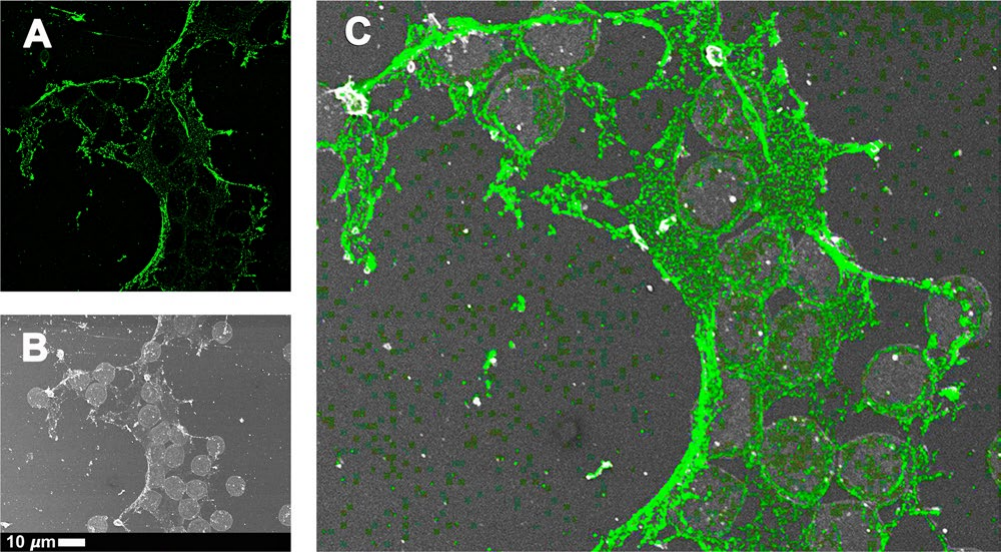
Representative (**A**) super-resolution (SR-SIM), (**B**) SEM and (C) CLEM micrographs of freshly-collected whole blood (WB) from a Parkinson’s Disease (PD) individual (1:200 dilution for primary and secondary antibodies). LPS antibody staining is closely associated with the fibre-like structures. Micrograph C colour was enhanced for publication clarity, by adjusting the vibrancy, brightness and contrast in Adobe Photoshop CS6.

CLEM, which allows the overlay of a confocal or super-resolution (fluorescence) micrograph onto a scanning electron microscope micrograph, by using a shuttle-and-find technique, was employed in order to indicate the location of the fluorescence LPS signal on the actual ultrastructure of the samples analysed. The fluorescence signal shows the presence of LPS in the PPP or WB, by direct antibody binding, while SEM shows the ultrastructure of the fibrin(ogen) or cellular structure. CLEM analysis therefore pinpoints the antibody-bound fluorescence signal to a specific area on the ultrastructure. Importantly, SR-SIM allows a higher resolution micrograph than confocal microscopy. In the CLEM analysis, we found that the fluorescence LPS signal is merged and fused into the dense matted fibrin(ogen) deposits, and we suggest that this incorporation of LPS into the fibrin(ogen) strands is an additional spatial and visual confirmation of the extent to which LPS is the causative agent of the presence of amyloid in inflammatory conditions such as PD, AD and T2D. Figure 8A shows a correlative micrograph of confocal and SEM micrographs overlaid of PPP from a T2D patient, while a correlative micrograph of SR-SIM and SEM micrographs overlaid of freshly-collected WB from a PD patient are shown in Fig. 8B. Due to the limit of resolution with confocal microscopy, the localisation of LPS seemed to be extended beyond that of the fibres (see Fig. 8A). With the improved resolution of SR-SIM, we showed that the LPS signal clearly correlates with the fibre structures, showing that LPS is closely associated with the actual fibres (see also Fig. 9). Citrated WB from a PD patient was also analysed (Fig. 8C). The storage method of the sample caused cell lysis and only cellular remnants are thus visible. Correlation of confocal and SEM micrographs illustrates that LPS antibody staining localises with the cellular content. Furthermore, SR-SIM, SEM and correlated micrographs of freshly-collected WB from a PD patient show that the fibrin(ogen) fibres are forming a network between and on top of the red blood cells (Fig. 9). The nature of the fibrin(ogen) fibres demonstrates hypercoagulability, with LPS antibody staining located in the same places as where amyloid fibrils may be observed. The binding of the antibody to fibre-like structures suggests that LPS plays a role in anomalous blood clotting.

## Discussion

Previously, we showed that the addition of highly substoichiometric levels of LPS caused fibrin(ogen) proteins in whole blood and platelet poor plasma from healthy individuals to polymerize into an amyloid form, with a greatly increased amount of ß-sheets. Healthy fibrin(ogen) protein structure shows more α-coils and fewer ß-sheets, but the addition of LPS changes the protein conformation^37–39^. This structural change both causes and reflects pathological clotting. We have also shown that in conditions like PD, AD and T2D, where there is an intrinsic, increased presence of circulating inflammagens, and an upregulation of inflammatory biomarkers (including various pro-inflammatory cytokines), pathological clotting is present^30,36,38,49,50,56,58,70^. We could show that such aberrant clotting is accompanied by amyloid formation in the fibrin(ogen) of these patients, and that this aberrant clotting can be reversed by adding LPS-binding protein^57–59,69^. In this paper, we confirmed the presence of increased amyloid in PD plasma (Fig. 3).

Extremely low levels of LPS can thus cause a cascade of events that lead to pathological clotting, and in this paper we sought to use a direct fluorescence LPS antibody-based technique to detect the presence of LPS in PD, AD and T2D. We found increased binding of antibody compared to controls. The binding of the antibody to fibre-like structures in the clots was further investigated using CLEM technology. With CLEM, it was confirmed that LPS strongly associated with all fibres in the field of view (Figs 8 and 9). This is the first use of such correlative microscopy to investigate the presence and location of LPS in WB and PPP. Super-resolution microscopy (Fig. 9), with its improved resolution, showed that the LPS signal clearly correlates with the fibre structures.

The specificity of antibodies is always a cause for concern^71,72^. However, cross-reactivity of the anti-*E*.*coli* LPS antibody (ab35654) was absent or minimal, as evident from the healthy controls in our experiments, as little LPS presence was detected. Because anti-*E*. *coli* LPS (ab35654) is a mouse monoclonal antibody, it will detect a specific epitope. Results obtained by Zhan and co-workers in 2016 also suggest little cross-reactivity with human proteins^73^. They found that LPS colocalized with Aβ_1–40/42_ in amyloid plaques and with Aβ_1-40/42_ around vessels in AD brains. DNA sequencing *confirmed E*. *coli* DNA in human control and AD brains^73^. Furthermore, based on the staining pattern found in rat intestine, this antibody does not seem to cross-react with other mammalian membrane proteins (see website link: http://www.abcam.com/E-coli-LPS-antibody-2D71-ab35654/reviews/24711). Grover and co-workers in 2012 also used ab35654 on yeast lysates and found no cross-reactive band in these cells^74^. Considering this, we suggest that our LPS antibody binding results are not influenced by cross-reactivity issues.

In conclusion, we showed that LPS is present in PPP and WB of patients with PD, AD and T2D; most importantly CLEM confirmed that it is closely associated in the fibrin fibre strands. Therefore we suggest that LPS could (at least in part) be a primary causative agent of abnormal clotting in PD, AD and T2D. This is a further confirmation that bacterial inflammagens can fuel the inflammatory processes in both systemic and neuroinflammatory conditions. It also implies strongly that decreasing (or removing completely) the availability of ‘free’ LPS could provide significant clinical and therapeutic benefits.

## Data Availability

Raw data, including original micrographs can be accessed at: https://1drv.ms/f/s!AgoCOmY3bkKHiM5kihHc4y7AfUm4HQ and on https://www.researchgate.net/profile/Etheresia_Pretorius.
